# Identification of Genomic Regions Associated with Powdery Mildew Resistance in Watermelon through Genome-Wide Association Study

**DOI:** 10.3390/plants13192708

**Published:** 2024-09-27

**Authors:** Oak-Jin Lee, Koeun Han, Hye-Eun Lee, Hyo-Bong Jeong, Nari Yu, Wonbyoung Chae

**Affiliations:** 1 Vegetable Research Division, National Institute of Horticultural and Herbal Science, Rural Development Administration, Wanju 55365, Republic of Korea; hke1221@korea.kr (K.H.); helee72@korea.kr (H.-E.L.); bong9846@korea.kr (H.-B.J.); ynr7328@korea.kr (N.Y.); 2 Department of Environmental Horticulture, Dankook University, Cheonan 31116, Republic of Korea; wbchae75@dankook.ac.kr

**Keywords:** watermelon, powdery mildew, genome-wide association study, germplasm collection, homozygous inbred line

## Abstract

Watermelon (*Citrullus* spp.) is an economically important crop globally, but it is susceptible to various diseases, including powdery mildew. Previous studies have identified genetic factors associated with powdery mildew resistance. However, further research using diverse genetic approaches is necessary to elucidate the underlying genetic mechanisms of this resistance. In this study, the germplasm collection comprising highly homozygous inbred lines was employed, which enabled the accumulation of consistent data and improved the reliability of the genome-wide association study (GWAS) findings. Our investigation identified two significant single-nucleotide polymorphisms (SNPs), *pm2.1* and *pm3.1*, which were strongly associated with disease resistance. Moreover, several candidate genes were revealed within the linkage disequilibrium (LD) blocks surrounding the significant SNPs. In conclusion, the identification of significant SNPs and their additive effects, combined with the discovery of relevant candidate genes, expanded our understanding of the genetic basis of disease resistance and can pave the way for the development of more resilient watermelon cultivars through marker-assisted selection.

## 1. Introduction

Watermelon (*Citrullus* spp.) is an economically important crop globally, accounting for 8.01% of agricultural production value among fruit worldwide, but it is susceptible to various diseases [1,2]. Powdery mildew, caused by the fungus *Podosphaera xanthii*, is a common fungal disease that affects watermelon plants. It causes powdery white spots to appear on the leaves, stems, and fruits [3]. This disease can lead to stunted growth, reduced fruit quality, and significant yield losses. Understanding the genetic basis of powdery mildew resistance in watermelon is crucial for developing effective and sustainable disease management strategies. In recent years, there has been a growing interest in understanding the genetic basis of the powdery mildew resistance in watermelons. Identifying the genes and mechanisms involved in resistance can lead to the development of resistant cultivars through breeding or biotechnological approaches [4,5,6,7,8,9].

Developing resistant varieties of watermelon is crucial for ensuring sustainable and productive cultivation. Resistance to powdery mildew and other diseases can significantly reduce the reliance on chemical pesticides, leading to more environmentally friendly and economically viable crop production. Additionally, resistant varieties can help to maintain consistent yields and improve fruit quality, even under challenging environmental conditions. By understanding the genetic basis of resistance and utilizing this knowledge in a breeding program, we can work toward developing watermelon varieties that are more resilient and have a higher tolerance to powdery mildew and other diseases.

The genome-wide association study (GWAS) has emerged as a powerful tool for dissecting the genetic architecture of complex traits, including disease resistance in crops. A GWAS allows for the identification of genetic markers associated with specific traits, providing valuable insights into the underlying genetic variation that contributes to the traits of interest. By analyzing diverse germplasm and conducting association mapping, a GWAS enables the identification of candidate genes and genomic regions linked to powdery mildew resistance, paving the way for targeted breeding efforts [10]. Genotyping by sequencing (GBS) offers a high-throughput and cost-effective approach for genotyping a large number of samples, facilitating the identification of genetic variants associated with a trait of interest. Through the integration of GBS data with phenotypic resistance, the breeding process is accelerated. The combined use of GWAS and GBS presents an opportunity to unravel the genetic basis of powdery mildew resistance in watermelons and can expedite the development of resistant varieties through marker-assisted selection [11].

Recent studies have clarified the genetic mechanisms underlying resistance to powdery mildew in watermelons. The inheritance pattern of resistance to race 1 W has been identified as being governed by three complementary and partially dominant genes [12]. In contrast, resistance to race 2 W appears to be regulated by a single dominant gene in PI 189225 [4]. Research involving other PIs has suggested a polygenic control of resistance or a mix of recessive and dominant genes [4,5]. Further studies have been conducted to identify the resistance genes. Kim et al. confirmed the genetic inheritance of resistance to race 1 W from the cultivar ‘Arka Manik’, which is controlled by a single incompletely dominant gene [6]. Moreover, they identified several candidate genes, including *Cla019831* and *Cla019844,* on Chromosome 2 using quantitative trait loci analysis and RNA sequencing. Additionally, Mandal et al. supported the findings of previous studies using differentially expressed gene (DEG) analysis, identifying *ClaPMR2* as an associated loci for powdery mildew resistance in watermelons, which is located on *Cla019831* [7]. A more recent study identified *ClLOX*, which encodes a lipoxygenase gene and is involved in inhibiting pathogen spread [8]. 

However, regarding the inheritance pattern, more than one gene might be involved in conferring resistance. Wu et al. identified several single-nucleotide polymorphisms (SNPs) significantly associated with powdery mildew 2 W resistance on 9 chromosomes, except Chromosomes 5 and 11, via a GWAS using the historic powdery mildew screening data of 1365 PIs [9]. Through bioinformatic analysis, Porterfield and Meru identified the chromosomal distribution of candidate susceptibility genes, including 14 mildew-locus-O (MLO) and 16 powdery mildew resistance (PMR) homologs, in watermelons [13]. Another comparative transcriptome analysis revealed several candidate genes involving phytohormone signaling and the phenylpropanoid pathway, and they were located on all chromosomes except Chromosome 8. 

Although a number of genetic factors associated with powdery mildew resistance have been identified in previous studies, research employing various genetic approaches are still necessary to elucidate the underlying genetic mechanisms of resistance [11]. In this study, we aimed to explore novel genetic regions and candidate genes associated with powdery mildew resistance in a collection of *Citrullus* spp., including wild-type germplasm. To date, there has been no prior research using GWASs on inbred lines for powdery mildew resistance. We utilized a germplasm collection composed of homozygous inbred lines, which facilitates clearer identification and analysis of the genetic factors influencing resistance. These results will be a useful resource in identifying key factors for powdery mildew resistance and will contribute to the development of more resilient watermelon varieties.

## 2. Results 

### 2.1. Identification of Genome-Wide SNPs in Watermelon Germplasm Collection

**Figure 1:**
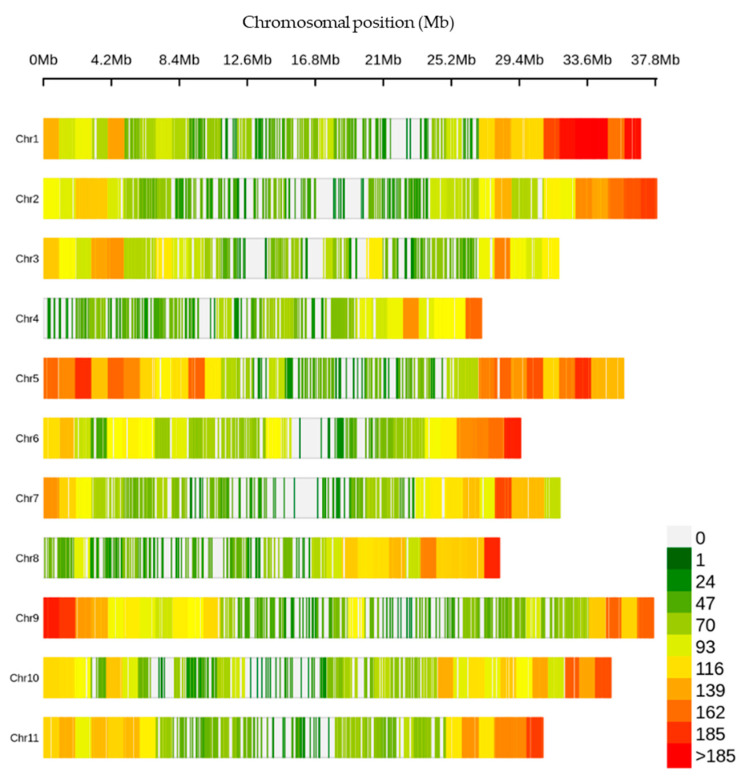
Density of the single-nucleotide polymorphisms (SNPs) across the chromosomes within a 1 Mb window size.

The 330,909 raw variants investigated in this study were identified through the SNP calling process. After applying the filtering criteria described in the materials and method, a final set of 29,765 high-confidence SNPs were identified across the genome of the germplasm collection. The number of SNPs per chromosome ranged from 1787 (Chromosome 4) to 3685 (Chromosome 5) (Table S1). The distribution of these SNPs was found to be uneven over the chromosomes, with Chromosome 5 exhibiting the highest number of SNP at an average interval of 10 kb and a maximum interval of 480 kb (Figure 1, Table S1).

### 2.2. Disease Resistance in the Watermelon Germplasm Collection

**Figure 2:**
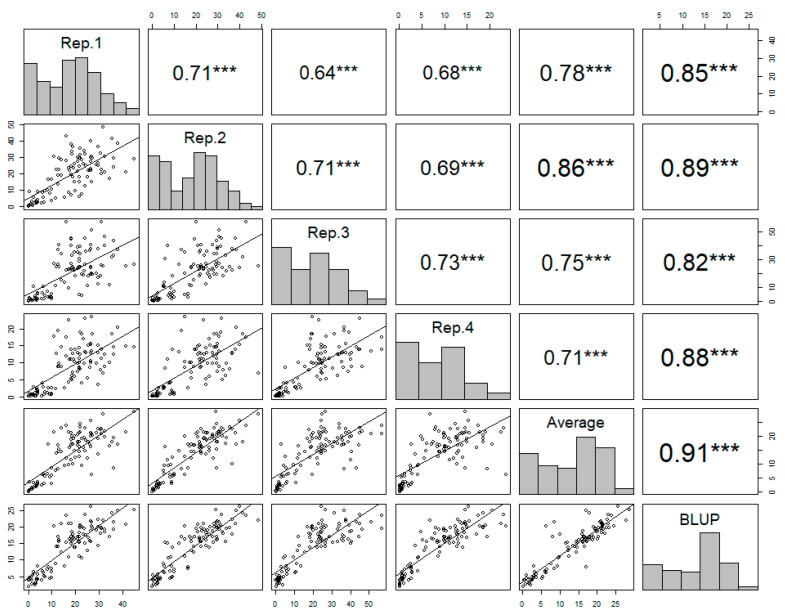
The correlation matrix of the four replicates, the averages of the replicates, and the best linear unbiased prediction (BLUP) values of each accession in powdery mildew resistance. Pearson’s correlation coefficients are indicated with asterisks, where *** denotes a *p* value less than 0.001, which indicates a highly significant correlation between the variables.

The observed phenotypic data showed a high level of consistency across the four replicates, confirming the reliability of the disease resistance of each accession. The Pearson’s correlation coefficients ranged from 0.71 to 0.73, indicating a moderate positive correlation across the replicates (Figure 2).

The best linear unbiased prediction (BLUP) values of the disease resistance were calculated for each accession based on the four replicates. The BLUP value showed a strong positive correlation with the average from the four replicates (Figure 2). The BLUP values reliably represented the phenotypic variance across the germplasm collection, accurately capturing the true genetic potential for disease resistance. The distribution of BLUP values across the germplasm collection displayed wide variation, from 2.05 to 26.03, indicating genetic diversity with regard to disease resistance (Figure 2).

### 2.3. Population Structure of the Germplasm Collection

**Figure 3:**
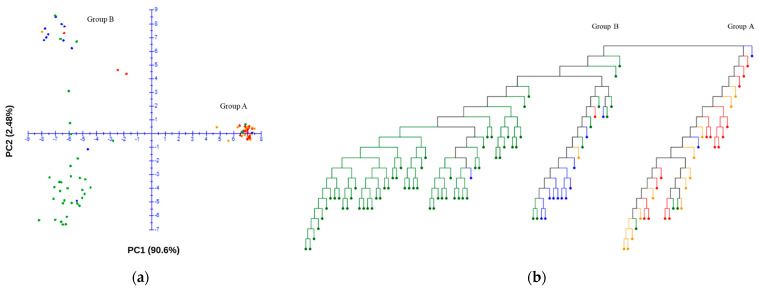
Principal component analysis (PCA) plot (**a**) and neighbor-joining tree (**b**) of the germplasm collection. The red and orange dots indicate the resistant and susceptible wild-type accessions, respectively. The blue and green dots indicate the resistant and susceptible domesticated-type accessions, respectively.

Using the final SNP dataset, principal component analysis (PCA) was employed to explore the genetic structure of the germplasm collection to indicate the existence of genetic diversity and control for potential influences from population stratification. The analysis included the genomic data of 109 accessions, and a comprehensive set of 29,765 SNPs were utilized after quality control. The PCA scatter plot displays the distribution of the first two principal components, which together account for 93.1% of the variance within the dataset. The germplasm collection was separated with the first and second principal components PC1 (90.60) and PC2 (2.48) (Figure 3a). A neighbor-joining dendrogram was constructed to visualize the genetic relationships among accessions (Figure 3b). Distinct clusters were observed in the PCA plot and neighbor-joining tree, where Group A, comprising approximately one third of the samples (32 accessions), was markedly separated from Group B. Group A primarily consisted of wild-type accessions, with 17 resistant and 15 susceptible accessions within this group, indicating a nearly equivalent distribution of powdery mildew resistance (Figure 3b). In contrast, Group B was predominantly composed of domesticated type accessions, with only two accessions that did not fit this classification. In this group, the resistant accessions are positioned in the upper quadrant of the PCA plot, while the susceptible accessions are clustered in the lower quadrant (Figure 3a). 

**Figure 4:**
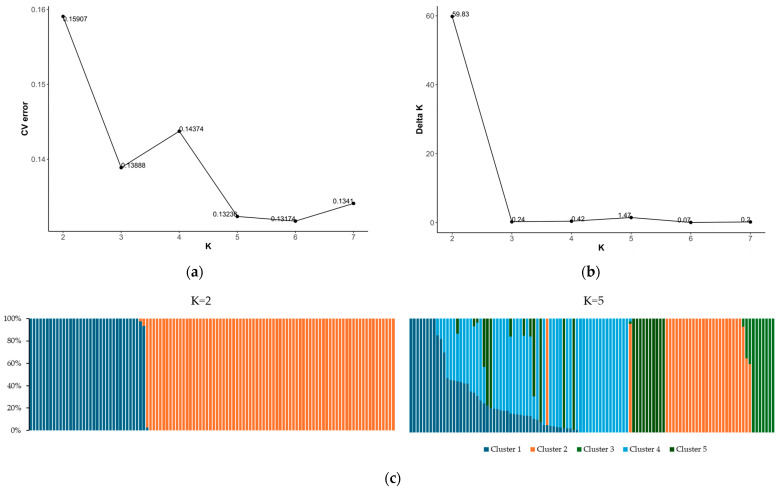
Population structure of the germplasm collection: (**a**) cross-validation (CV) error plot; (**b**) delta K plot; and (**c**) population clustering patterns for informative K values (K = 2 and K = 5).

Building on the insights gained from PCA, we further explored the population structure using ADMIXTURE analysis to assess the degree of genetic admixture within the germplasm collection. The best K was determined using the delta K method, which indicated a clear peak at K = 2 (Figure 4b). This suggests a strong signal for the two main genetic clusters within the population. This was a consistent result with the PCA, which showed the separation along the first principal axis. The cross-validation (CV) error plot showed a continuous decrease as K increased, with the lowest error at K = 5, suggesting the presence of a substructure within the previously identified clusters (Figure 4a). Despite the lower CV error observed at K = 5, the sharp peak in the delta K at K = 2 was considered the primary determinant for the number of clusters due to its robust methodological preference in detecting the true number of clusters. Therefore, the population structure was primarily divided into two genetic clusters, which aligned with the major separations observed among the accessions. Cluster 1 predominantly comprised wild-type accessions, which accounted for 32% of the germplasm collection. Cluster 2, which included mostly domesticated accessions, represented approximately 68% of the population (Figure 4c).

The polymorphism information content (PIC) values of the final SNPs showed significant polymorphism, with an average of 0.989, indicating a strong genetic diversity in the germplasm collection and suitability for GWAS. Most markers had a high discriminatory power, making them well suited for identifying the genetic associations with specific phenotypic traits across diverse populations. These results support the subsequent analyses of genetic association and the identification of genotype–phenotype correlations within this population (Figure S1).

Considering the observed population structure and genetic variation within the germplasm collection, we adjusted the GWAS by incorporating the first two principal components as covariates. This adjustment aimed to minimize the potential biases due to population structure and more accurately identify the true associations between the traits of interest and genetic variants. Additionally, complex linear models were applied to adjust for possible biases due to kinship relationships [14,15].

### 2.4. Genome-Wide Association Study

Our comprehensive GWAS aimed to elucidate the genetic underpinnings of the phenotypic variation in the resistance to powdery mildew within the germplasm collection. The generalized linear model (GLM), a mixed linear model (MLM), and a Bayesian-information and linkage-disequilibrium iteratively nested keyway (BLINK) model were employed to account for the population structure and kinship results. We utilized a set of 29,765 high-quality SNPs distributed across the genome in the germplasm collection.

**Figure 5:**
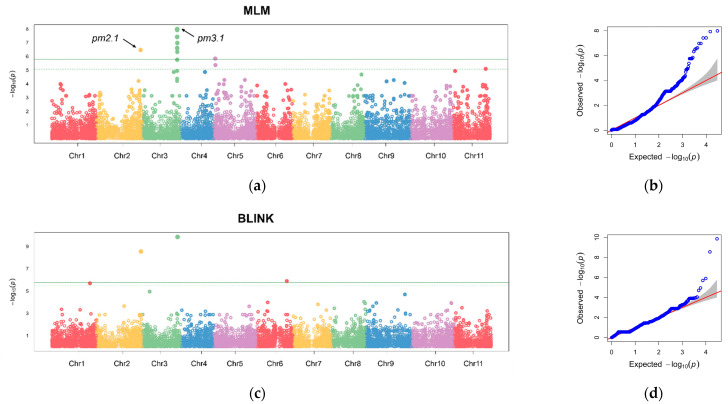
Manhattan plots and quantile–quantile (Q-Q) plots from a genome-wide association study (GWAS) of the best linear unbiased prediction (BLUP) values of each accession in powdery mildew resistance. (**a**,**b**) Manhattan plot and Q-Q plot using a mixed linear model (MLM). (**c**,**d**) Manhattan plot and Q-Q plot using a Bayesian-information and linkage-disequilibrium iteratively nested keyway (BLINK) model. The horizontal solid and dashed lines indicate the genome-wide and suggestive significance thresholds, respectively. Each chromosome is colored differently. The red line represents the expected distribution under the null hypothesis, where no association exists. The grey area indicates the 95% confidence interval.

The GLM identified several genomic regions on Chromosomes 2, 3, 4, 5, 9, and 11, which were significantly associated with disease resistance across the four replicates and BLUP values consistently (Figure S2). Further investigation was conducted using the MLM and BLINK models with the BLUP values to refine the association results [14,15]. Our rigorous statistical models revealed 11 and 3 significant SNPs that surpassed the stringent 1% Bonferroni correction threshold for MLM and the BLINK model, respectively (Figure 5). The most robust association was observed on Chromosome 3 at Position 29,021,875, designated as *pm3.1*. Another notable locus was identified on Chromosome 2 at Position 36,647,891, designated as *pm2.1*.

The significant SNPs accounted for 33.3% and 23.3% of the phenotypic variance, respectively, for *pm2.1* and *pm3.1* according to the BLINK model (Table 1). The analysis revealed that *pm2.1* exhibited a positive effect size of +3.82, implying that the presence of alternative allele is correlated with an increase in the infected leaf area (Table 1). This association points to a potential risk factor for the susceptibility among germplasm collection, which has the alternative allele on *pm2.1*. On the contrary, *pm3.1* is associated with a negative effect size of −5.01 (Table 1). This finding suggests that the alternative allele on *pm3.1* might contribute to a decrease in the infected leaf area, indicating a possible protective role against the pathogen. The minor allele frequency (MAF) of the *pm2.1* was 0.1071, indicating that the major allele associated with reduced infected leaf area was present in approximately 89.3% of the germplasm collection. In contrast, the allele frequency at the *pm3.1* was 0.2232, implying that the minor allele linked to increased resistance is less common in the population (Table 1). The higher frequency of the resistance allele at the *pm2.1* suggests that it may have a more widespread influence on the resistance across the population compared to the *pm3.1*. As such, while the *pm3.1* may contribute additional factors that enhance disease resistance, its overall impact is likely less pronounced than that of the *pm2.1*.

### 2.5. Genotypic and Phenotypic Variation of the Significant SNPs in the Germplasm Collection

Out of the 109 total accessions, 97 had the resistance allele for *pm2.1*, while only 22 had the resistance allele for *pm3.1*. Among the accessions with the *pm2.1* resistance allele, 77 out of 80 (96.3%) were identified as domesticated type accessions, and 20 out of 29 (69.0%) were wild-type accessions. Only 3 out of 80 (3.8%) domesticated type accessions possessed the *pm3.1* resistance genotype, whereas 19 out of 29 (65.5%) wild-type accessions carried it. The findings suggest that the domesticated type accessions exhibited a markedly higher proportion of the *pm2.1* resistance, while the wild-type accessions demonstrated a substantially greater frequency of the *pm3.1* resistance genotype.

**Figure 6:**
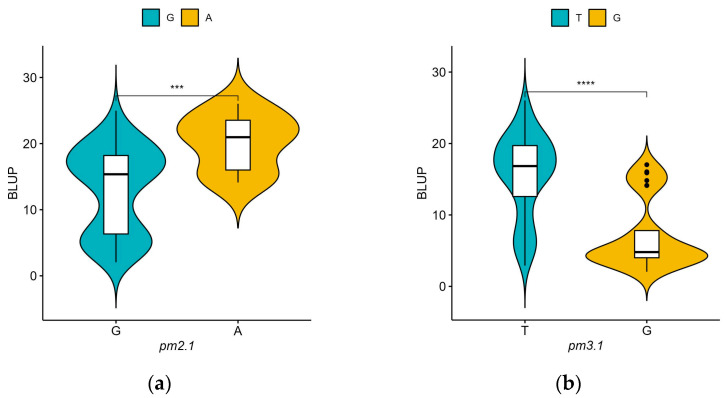
The phenotypic distribution of the best linear unbiased prediction (BLUP) values of each accession in powdery mildew resistance by allele for significant single-nucleotide polymorphisms (SNPs): *pm2.1* (**a**) and *pm3.1* (**b**). Blue and yellow represent the reference and alternative alleles, respectively. The asterisks denote the four levels of significant differences from the *t*-test results (**** *p* < 0.0001, *** *p* < 0.001).

**Figure 7:**
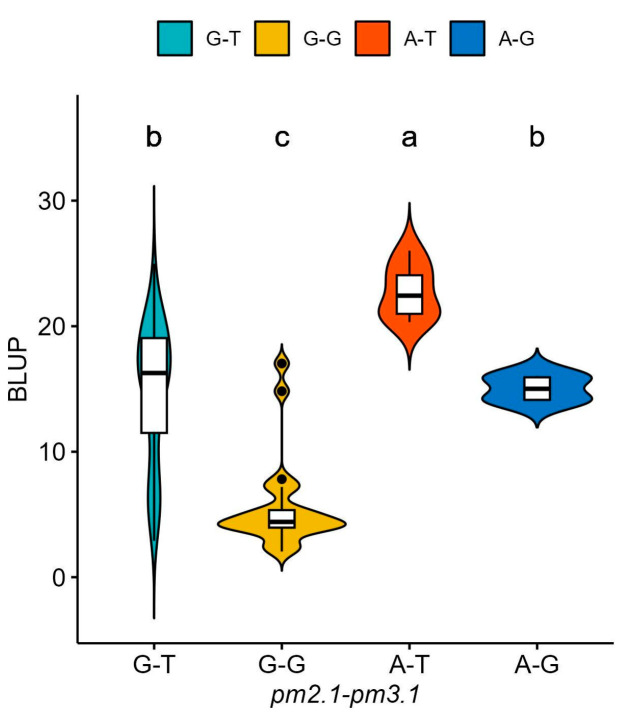
The phenotypic distribution of the best linear unbiased prediction (BLUP) values of each accession in powdery mildew resistance based on the allelic combinations of the significant single-nucleotide polymorphisms (SNPs): *pm2.1* and *pm3.1*. Different small letters refer to significant differences according to Duncan’s multiple range test.

**Figure 8:**
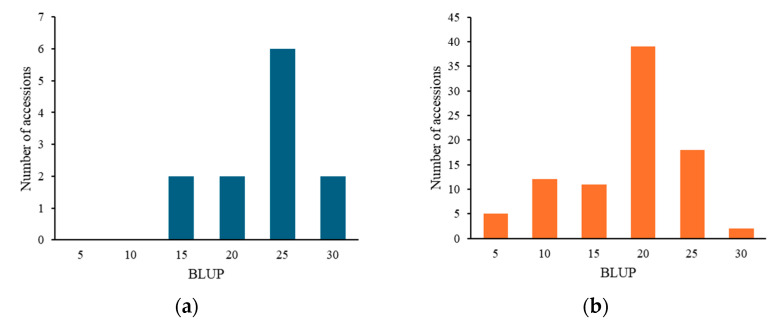
The distribution of the best linear unbiased prediction (BLUP) values across the accessions with susceptible alleles on *pm2.1* (**a**) and *pm3.1* (**b**).

For *pm2.1*, the analysis showed that the G alleles were associated with higher resistance indicating a median BLUP value of 15 (Figure 6a). In the case of *pm3.1*, accessions with the G alleles exhibited lower BLUP values, with a median around 5, which is indicative of higher disease resistance (Figure 6b). Accessions that had resistance genotypes for both SNPs were more frequently found in the wild-type accessions with 16 out of 29 (55.2%) accessions when compared to the only 2 out of 80 (2.5%) in the domesticated type accessions. Most of them showed high disease resistance in the bioassay no matter which species they were. According to the phenotypic distribution of the germplasm collection, accessions with the G-G genotype allele at significant SNPs exhibited the highest disease resistance. Conversely, the A-T genotype showed considerably lower resistance (Figure 7). When *pm2.1* exhibited the susceptible genotype, the accessions showed higher than 15 in BLUP value regardless of the *pm3.1* genotype (Figure 8). Among the samples of which *pm3.1* was the susceptible genotype, some accessions exhibited a certain level of resistance, i.e., lower than 10 in BLUP value, if *pm2.1* was the resistance genotype (irrespective of the species).

**Figure 9:**
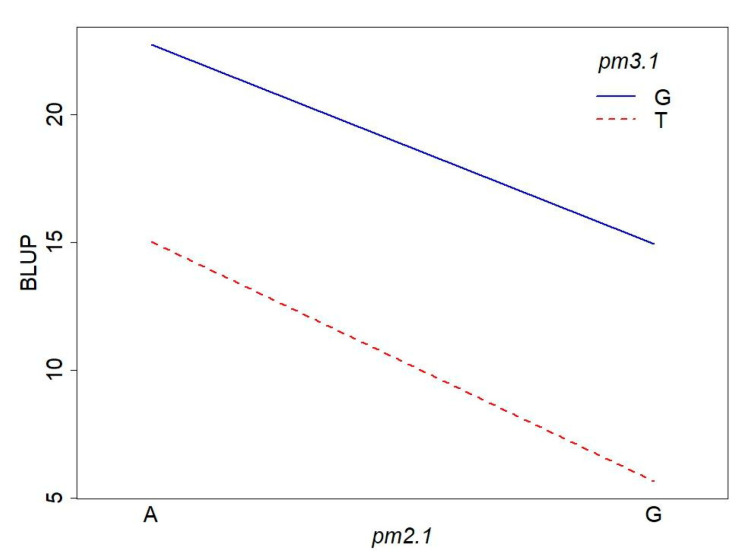
Interaction plot of the *pm2.1* and *pm3.1* genotypes on powdery mildew resistance.

To investigate the interaction effect of two SNPs, we conducted a two-way ANOVA on disease resistance. The ANOVA results revealed that both *pm2.1* and *pm3.1* had significant main effects on the powdery mildew resistance (Table 2, Figure 9). The effect of *pm2.1* was highly significant, indicating that different genotypes at this locus contribute to variations in the trait. Similarly, *pm3.1* also showed a highly significant effect, suggesting a strong association between the SNP and the trait. Interestingly, the interaction effect between *pm2.1* and *pm3.1* was not significant (Table 2, Figure 9). This indicates that the effect of one SNP on the resistance does not depend on the genotype of the other SNP. In other words, the contribution of *pm2.1* to the resistance is consistent across different genotypes of *pm3.1*, and vice versa. 

### 2.6. Candidate Genes for Powdery Mildew Resistance 

To investigate whether the significant SNPs identified through the GWAS had the potential to alter gene function, we employed the snpEff program to annotate and predict the effects of the genetic variants. *pm2.1* was located on the ABC transporter domain-containing protein, resulting in a missense variant. This suggests that *pm2.1* causes an amino acid substitution in the protein, potentially altering its function or stability. *pm2.1* was also positioned upstream of the gene encoding the ABC transporter G family member 15-like. This upstream location may influence the transcriptional regulation of the protein, potentially affecting its expression levels. *pm3.1* was located downstream of the gene encoding mitogen-activated protein kinase kinase 2 and serine/arginine repetitive matrix protein. While this downstream position is less likely to affect the coding sequence of the proteins, it might influence the regulatory elements or gene expression levels.

Further analysis was conducted on the adjacent regions of the identified SNPs [16]. The size of the adjacent region was defined as 100 kb upstream and downstream of each significant SNP according to the average of the linkage disequilibrium (LD) block size in watermelons described by Nimmakayala et al. [17]. Each of these genes became a focal point for subsequent scrutiny as we aimed to elucidate their potential contributions to the observed powdery mildew resistance. This investigation unveiled several potential genes that may play a role in powdery mildew resistance. Around *pm2.1* and *pm3.1*, there were 56 genes including those encoding unknown proteins. Upon querying the candidate genes on the adjacent regions of the identified SNPs in the UniProt and plant resistance gene database (PRGdb) 4.0, several proteins were identified as being related to the plant defense and/or innate immune response mechanisms in plants (Table 3) [18,19]. There were no proteins detected that may have been involved in the plant susceptibility near SNPs such as MLO-like proteins [13,20].

## 3. Discussion

In this study, the germplasm collection was notably composed of inbred lines characterized by high levels of homozygosity. Utilizing a stable genetic background not only enables the accumulation of repeated data, but also improves the reliability of GWAS results, enhancing the statistical power of a study. This genetic uniformity ensured that the observed phenotypic variations were attributable to genetic differences rather than heterozygosity, thereby simplifying the identification of trait-associated alleles. From this diverse yet genetically refined pool, 109 accessions exhibiting pronounced phenotypic diversity for the disease resistance were meticulously selected for in-depth genetic analysis.

The BLUP values, which account for both the fixed and random effects, provided a refined assessment of the genotypic effect on disease resistance [22]. These values enabled a more accurate estimation of the genetic potential of each accession in the absence of environmental and experimental noise. This selection approach driven by BLUP values ensures that a GWAS covers a wide range of genetic diversity, increasing the chances of identifying the important genetic markers linked to disease resistance.

In our GWAS, we identified significant SNPs located on Chromosomes 2 and 3. The allele at the *pm2.1* locus, which confers disease resistance, was identified as the major allele in our population. The effect size associated with this locus had a positive value, suggesting that the presence of this allele is beneficial in reducing disease symptoms. Conversely, the occurrence of an alternative allele at this locus led to an increase in disease severity, as reflected by a higher infected area. The dominance of the major allele in the population can be attributed to the strong selective pressure favoring disease resistance, which is crucial for the survival and productivity of a crop. In contrast, the allele at the *pm3.1* locus, which also confers disease resistance, was identified as a minor allele in our population. The effect size at this locus exhibited a negative value, indicating that the presence of the alternative allele at the *pm3.1* locus resulted in a reduction in the infected area, thereby enhancing the disease resistance. The MAF suggests that the beneficial allele may be subject to distinct selective pressures compared to the allele at *pm2.1*. This could potentially be attributed to inherent genetic factors, such as its origin from wild-type germplasm, which may influence its prevalence within the population. It suggests the complexity of the genetic contributions to the powdery mildew resistance, with both protective and risk factors identified through the GWAS approach. The contrasting directions of effect sizes for *pm2.1* and *pm3.1* underscore the intricate genetic interplay involved in resistance mechanisms. The analysis further indicates that there is no interaction effect between *pm2.1* and *pm3.1*. This means their influence on disease resistance is independent of each other, thus allowing the effect on disease resistance to be considered as a simple additive effect of the two SNPs. In conclusion, for molecular breeding programs aimed at improving disease resistance, selecting for the alternative allele of *pm2.1* and the reference allele of *pm3.1* offers a strategic advantage. These findings have important implications for molecular breeding programs. The significant main effects of both SNPs suggest that they could be valuable markers for selecting desirable traits in breeding populations.

Previous studies have identified candidate genes associated with powdery mildew resistance on Chromosome 2, especially within the 29.1 Mb to 34.8 Mb region [6,7,8]. Among these studies, *Cla019831* was proposed as a potential resistance gene, suggesting its significant role in conferring resistance in *C. lanatus*. However, the resistance-associated region on Chromosome 2 was located around the 36.6 Mb region in our study, which is slightly different with the previously reported candidate region and did not include *Cla019831*. This discrepancy could be attributed to the inclusion of wild-type accessions in the germplasm collection. The genetic diversity present in wild-type accessions may have introduced novel alleles or loci that are not represented in domesticated populations, leading to the identification of a distinct resistance-associated region [23]. Additionally, it highlights the inherent differences between a GWAS and quantitative trait loci (QTL) mapping analysis. In genetic research, QTL mapping and GWAS employ distinct statistical methods and assumptions, which can lead to different findings [24]. QTL mapping, using linkage analysis in a specific breeding population, focuses on how genetic variations linked to traits are inherited, emphasizing closely related genetic markers. Conversely, a GWAS explores broader correlations across diverse populations using association analysis, which assesses widely dispersed genetic markers. Additionally, a GWAS employs association analysis to explore the correlations across a broad natural population, and QTL mapping utilizes linkage analysis within specific breeding populations. This divergence in methodologies may result in inconsistencies in genomic marker identification, as demonstrated by the differing results for *pm2.1* and *pm3.1*. Additionally, the use of GBS in our study presents limitations that could impact the representation of certain genomic regions [25]. This specific approach only sequences at certain restriction sites, potentially omitting significant regions from analysis, as what had possibly occurred with the previously reported candidate regions in our GWAS.

There were eight candidate genes in the LD block of the significant SNPs. These included calcium-dependent protein kinase, mitogen-activated protein kinase kinase 2, and serine/threonine–protein kinase tricorner, which are the putative plant disease resistance genes orthologs collected from the National Center for Biotechnology Information (NCBI) and computationally predicted on PRGdb 4.0 [19]. Through evolutionary adaptation, plants have acquired the capacity to recognize pathogens and subsequently activate defense mechanisms [3]. These defense responses are initiated by specific receptors encoded by pathogen recognition genes (PRGs). These genes can be categorized into different functional classes based on the presence of specific domains, such as leucine-rich repeats [19]. Calcium-dependent protein kinase (CDPK) contains only a transmembrane helix domain, which primarily functions in pathogen recognition at the cell surface, acting as a sensor that detects the presence of pathogens and relays this information across the cell membrane to initiate defense response [26]. Previous studies have demonstrated that specific paralogs exhibit antagonistic roles in controlling the host cell entry during powdery mildew infection in barley [27]. Similarly, in wheat, the CDPK is required for resistance to powdery mildew, as well as to bacterial blight, suggesting a broader role of the protein in plant immunity [28]. Further, the research on wild grapevine has revealed that the overexpression of CDPKs enhances powdery mildew resistance by upregulating salicylic acid and ethylene signaling pathways, which are essential components of the immune response [29]. On the other hand, mitogen-activated protein kinase kinase 2 (MAPKK2) and serin/threonine–protein kinase tricorner are PRGs that contain a kinase domain. They are involved in the intracellular signal transduction processes that follow pathogen recognition by phosphorylate specific target proteins, thereby modulating signaling cascades that lead to a variety of defense responses, such as the production of antimicrobial compounds and the activation of programmed cell death to contain the pathogen [26]. The mitogen-activated protein kinase signaling pathways, particularly those involving MAPKK2, have emerged as crucial regulators of plant stress responses, growth, and development [30]. Consistent with previous research, the findings of this study indicate that MAPKK2 plays a key role in both biotic and abiotic stress tolerance [30]. In *Arabidopsis thaliana*, MAPKK2 has been shown to function redundantly in mediating defense responses via jasmonic acid-dependent and salicylic acid-dependent pathways [30]. Furthermore, MAPKK2’s involvement in plant development is highlighted by its regulation of cellular processes such as hormone signaling and immune responses, suggesting a broader role in ensuring plant adaptability to varying environmental conditions [31]. A recent study identified serine/threonine–protein kinases, including CsGy5G015660, as key regulators of defense responses against powdery mildew in cucumbers, providing a genetic basis for breeding resistant varieties [32]. Additionally, studies on the defense mechanisms in cucumbers further support the role of these kinases in triggering resistance pathways, with the induction of serine/threonine–protein kinase kinases acting as positive regulators of immune responses against powdery mildew [33].

Further investigation using the UniProt database revealed additional putative candidate resistance genes, such as those encoding cinnamoyl-CoA reductase, RING-type E3 ubiquitin transferase, and DDE Tnp4 domain-containing protein [18]. Cinnamoyl-CoA reductase, an enzyme involved in the latter stages of lignin biosynthesis, has been implicated in plant defense in *Oryza sativa* subsp. *japonica* and *Arabidopsis thaliana* [34,35]. Specifically, cinnamoyl-CoA reductase 1 likely contributes to lignin formation during defense responses, while cinnamoyl-CoA reductase 2 may play a role in the production of phenolic compounds associated with the hypersensitive response in *Arabidopsis* [36]. Additionally, various subtypes of the RING-type E3 ubiquitin transferase enzyme have been shown to participate in plant defense mechanisms in *Arabidopsis*. The subtype ATL gene family can catalyze polyubiquitination, which is potentially involved in the early stages of defense signaling pathways [37]. Subtype ATL55 may contribute to the positive regulation of programmed cell death by facilitating the degradation of negative regulators [38]. Subtypes RIN3 and PUB23 act as regulators of the hypersensitive response in association with RIN2 [39,40]. Furthermore, there is a DDE Tnp4 domain-containing protein, which contains a central nucleotide-binding subdomain and a coiled-coil structure at the N-terminal region. This protein is a type of pathogen recognition gene called CC-NB-LRR and has been implicated in plant defense mechanisms in *Triticum aestivum* [18]. It acts as a receptor that detects specific effector proteins that are produced by pathogens, triggering a signaling cascade that activates the plant’s immune response [19]. Additionally, phenylalanine ammonia-lyase, an enzyme involved in the phenylpropanoid pathway, was found to be located near the *pm3.1*. This protein was identified as one of the differently expressed genes between resistant and susceptible lines of *C. lanatus* after inoculation [21]. They suggest that the phenylpropanoid pathway plays a key role in conferring local resistance against powdery mildew.

Previous studies have primarily focused on Chromosome 2, as described above. In this study, we identified a novel powdery mildew resistance-associated genetic region on Chromosome 3 that has not been previously reported. Additionally, the phenotypic variation depends on the genotypes revealed that the resistance genotype on *pm2.1* is essential for conferring baseline resistance. However, the presence of the resistance genotype on *pm3.1* is crucial for achieving a higher level of resistance. This might indicate a complementary interaction between these loci, where *pm2.1* provides the fundamental resistance mechanism, while *pm3.1* enhances the resistance through an additive effect. Moreover, the resistance genotype of *pm3.1* was more commonly found in the wild-type accessions. This finding not only broadens the scope of genetic regions associated with disease resistance, but also provides new targets for marker-assisted selection in breeding programs aimed at developing disease-resistant varieties, especially when we try to introduce the resistance from wild-type accessions [41]. The identification of the novel region and its associated SNPs paves the way for further functional studies to elucidate the underlying genetic mechanisms and to validate the use of these markers in practical breeding applications.

## 4. Materials and Methods 

### 4.1. Plant Materials and Growth Conditions

A total of 109 inbred lines of watermelon accessions were included in this study, and they were sourced from genetic materials provided by the National Plant Germplasm System (Washington, DC, USA) and Rural Development Administration-Genebank (Jeonju, Republic of Korea). The accessions were collected from diverse geographical regions, including Asia (53 accessions, 48.6%), Africa (48 accessions, 44.0%), and North America (7 accessions, 6.4%). Among the germplasm collection, 80 were domesticated-type accessions (*C. lanatus*) and 29 were wild-type accessions (*C. amarus* or *C. mucosuspermus*) (Table S2).

The seeds of selected accessions were planted in 50-cell trays filled with a standard horticultural soil mix (Heung Nong Co., Ltd., Pyeongtaek, Republic of Korea). The trays were initially placed in a dark incubation chamber kept at 28 °C and a 90% relative humidity for 2 days to promote consistent germination. After germination, the trays were transferred to a growth chamber where environmental conditions were controlled at 25/16 °C (day/night) with a 16 h photoperiod. When the seedlings developed two-to-three leaves, they were carefully translocated to new trays arranged in a grid pattern to ensure even exposure to the powdery mildew conidia one day before inoculation. 

### 4.2. Powdery Mildew Resistance Evaluation

The initial conidia of powdery mildew were collected from naturally diseased leaves in a greenhouse at the National Institute of Horticultural and Herbal Science (NIHHS) in Wanju, Korea. These conidia were regularly maintained on susceptible watermelon lines in the growth chamber every month. The suspension of conidia, concentrated on 1 × 10^5^ conidia per mL in 0.02% tween-20, was sprayed thoroughly onto the leaf surface. The severity of the powdery mildew infection on each seedling was assessed 13 days after inoculation as the percentage of leaf area with lesion. Each accession underwent four replicates to ensure the reliable observation for disease resistance assessment. To account for environmental variability and to enhance the accuracy of genetic effect estimation, BLUP values for each accession were calculated using the ‘lme4’ package version 1.1.34 in R, which is a comprehensive tool for fitting linear and nonlinear mixed-effects models. Additionally, Pearson’s correlation analysis was conducted to assess the similarity among replicates with the ‘PerformanceAnalytics’ package version 2.0.4 in R.

### 4.3. DNA Extraction

The fresh leaf tissue of each accessions was collected from the cotyledon before the inoculation. The collected samples were grounded into a fine powder in liquid nitrogen. Genomic DNA was extracted using a modified cetyltrimethylammonium bromide (CTAB) method [42]. The concentration and purity of the extracted DNA were assessed using a NanoDrop ND-2000 (NanoDrop Technology, Inc., Wilmington, DE, USA) and PicoGreen dsDNA assay (Quant-iT™ PicoGreen^®^ dsDNA Assay Kit, Thermo Fisher Scientific, Waltham, MA, USA) following the manufacturer’s instructions.

### 4.4. Genotyping by Sequencing and Single-Nucleotide Polymorphism Calling

The genomic DNA samples were processed for GBS analysis according to the protocol described by Elshire et al. [25]. Briefly, DNA samples were digested with the restriction enzyme ApeKI, and adapters containing unique barcodes were ligated to each sample. Pooled libraries were then sequenced on an Illumina NovaSeq 6000 platform (Illumina Inc., San Diego, CA, USA), generating over 400 Mb of reads per sample. SNP calling was performed to identify the genetic variations across the watermelon germplasm collection genomes. The raw reads obtained from GBS were aligned to the watermelon reference genome ‘97103 (*C. lanatus*)’ version 2 at the CuGenDB (http://cucurbitgenomics.org/; accessed on 27 August 2024) using a custom pipeline for quality control, including the trimming of adapter sequences and removal of low-quality reads [43]. The cleaned reads were aligned to the reference watermelon genome using the Burrows–Wheeler aligner (BWA) version 0.7.17. SNPs were called using the genome analysis toolkit (GATK) version 4.2.0.0, with stringent filtering criteria applied to select high-confidence SNPs. To ensure the reliability and accuracy of our SNP dataset, we implemented a SNP filtering criteria including a minimum read depth of 5x, a base quality score of >20, and a mapping quality score of >20. This process involved the removal of SNPs with a MAF below 5%, as well as those SNPs exhibiting more than 5% missing data across the samples.

### 4.5. Population Analysis

The PCA plot and neighbor-joining tree construction were performed using DARwin6 software version 6.0.21 [44]. The PCA plot was used to visualize the genetic diversity and relationships among the populations, while the neighbor-joining tree was constructed to infer the phylogenetic relationships based on genetic distances. The population structure was analyzed using the ADMIXTURE software version 1.3.0 [45]. The analysis was conducted under the assumption of different numbers of ancestral populations (K = 2 to K = 7). The CV error was calculated for each K value, and the optimal number of populations was determined based on the lowest CV error. PIC values were calculated using the pysam library version 0.22.0 in Python [46]. The VCF files containing variant information were processed to compute PIC values for each SNP. PIC values were used to assess the informativeness of the genetic markers within the population.

### 4.6. Genome-Wide Association Study

The high-quality, imputed SNP dataset served as the basis for a GWAS aimed at identifying SNPs associated with resistance to powdery mildew in watermelons. The GWAS analysis was conducted using the lesion area of powdery mildew and the calculated BLUP values as phenotypic data to identify the significant associations between SNPs and the traits of interest. Several approaches including the GLM, MLM, and BLINK model were employed. This was facilitated by the ‘GAPIT’ package version 3 in R, which were tailored for high-dimensional genetic data [47]. The results of the GWAS were visualized using Manhattan plots to highlight the significant SNP–trait associations, and quantile–quantile (Q-Q) plots were used to assess the overall significance level relative to the expected distribution under the null hypothesis. This comprehensive statistical framework, incorporating the calculation of BLUP values and GWAS, enabled the identification and characterization of genetic factors underlying the trait variation in watermelons. To visualize the distribution of the phenotypic values on the significant SNPs, violin plots were generated using the ‘ggplot2’ package version 3.5.1 in R. Furthermore, a two-way ANOVA was performed using R software to evaluate the interactions between the significant SNPs on the resistance. Significant variants were annotated using snpEff software version 5.0e to predict the functional effects [48]. Candidate genes associated with significant SNPs were investigated using the reference genome, Uniprot database, and PRGdb version 4.0 [18,19].

## 5. Conclusions 

In this study, we utilized 109 inbred lines of watermelon to conduct a GWAS aimed at identifying significant SNPs associated with powdery mildew resistance. Our GWAS results successfully identified two significant SNPs that exhibited a strong correlation with disease resistance. Further analysis of the genotypic combinations of these two SNPs demonstrated an additive effect on disease resistance, indicating the potential for combining these alleles to enhance the resistance in watermelon breeding programs.

Moreover, we performed a detailed search for candidate genes within the LD blocks surrounding these significant SNPs. This search revealed several candidate genes potentially involved in disease resistance mechanisms. These findings provide valuable insights into the genetic basis of the disease resistance in watermelons and highlight the specific genomic regions and candidate genes for further functional studies and breeding efforts.

In conclusion, the identification of significant SNPs and their additive effects, along with the discovery of candidate genes within relevant LD blocks, underscores the potential of molecular breeding strategies in improving the disease resistance in watermelons. These results contribute to the broader understanding of the genetic architecture of disease resistance and pave the way for the development of more resilient watermelon cultivars through marker-assisted selection.

## Figures and Tables

**Table 1:** Summary of the significant single-nucleotide polymorphisms (SNPs) identified through a GWAS on Chromosomes 2 and 3 in the germplasm collection.

SNP	Chromosome	Position (bp)	*p* Value	MAF ^1^	Effect Size	PVE (%) ^2^
*pm2.1*	2	36,647,891	2.82 × 10^−9^	0.1071	3.82	33.3
*pm3.1*	3	29,021,875	1.41 × 10^−10^	0.2232	−5.01	23.3

^1^ MAF, minor allele frequency. ^2^ PVE, phenotype variance explained.

**Table 2:** Results of the two-way ANOVA used to assess the interaction between the *pm2.1* and *pm3.1* genotypes on powdery mildew resistance. The asterisks denote the three levels of significant differences (*** *p* < 0.001 and ns means not significant).

Effect	Df ^1^	Sum Sq ^2^	Mean Sq ^3^	*F* Value	*p* Value	
*pm2.1*	1	554.082	554.082	22.457	6.59 × 10^−6^	***
*pm3.1*	1	1579.576	1579.576	64.021	1.50 × 10^−12^	***
*pm2.1*:*pm3.1*	1	5.755	5.755	0.233	0.6301	ns
Residuals	108	2664.674	24.673			

^1^ Df, degree of freedom; ^2^ Sum Sq, sum of squares; and ^3^ Mean Sq, mean square.

**Table 3:** Candidate genes associated with powdery mildew resistance in the adjacent regions of the significant single-nucleotide polymorphisms (SNPs) identified in the genome-wide association study (GWAS).

Gene ID	Chr.	Position (bp)	Description	Reference
Cla97C02G049140	2	36,631,353…36,634,962	Cinnamoyl-CoA reductase 1-like	[18]
Cla97C02G049220	2	36,688,474…36,691,628	Cinnamoyl-CoA reductase	[18]
Cla97C02G049250	2	36,706,805…36,709,671	Calcium-dependent protein kinase	[18,19]
Cla97C03G065500	3	28,932,041…28,932,574	RING-type E3 ubiquitin transferase	[18]
Cla97C03G065540	3	28,959,829…28,961,964	Phenylalanine ammonia-lyase	[21]
Cla97C03G065600	3	29,015,917…29,019,130	Mitogen-activated protein kinase kinase 2	[18,19]
Cla97C03G065640	3	29,034,873…29,042,615	Serine/threonine-protein kinase tricorner	[19]
Cla97C03G065660	3	29,048,112…29,049,554	DDE Tnp4 domain-containing protein	[18]

## Data Availability

The original contributions presented in this study are included in this article/Supplementary Material, and further inquiries can be directed to the corresponding authors.

## Supplementary Materials

The following supporting information can be downloaded at: https://www.mdpi.com/article/10.3390/plants13192708/s1, Figure S1. Distribution of the polymorphic information content (PIC) across single-nucleotide polymorphisms (SNPs). Figure S2. Manhattan plot from the genome-wide association study (GWAS) using a generalized linear model (GLM) for four replicates (a–d), the averages of the replicates (e), and the best linear unbiased prediction (BLUP) values (f) of each accession in powdery mildew resistance. The horizontal solid and dashed lines indicate genome-wide and suggestive significance thresholds, respectively. Table S1. Distribution of the single-nucleotide polymorphisms (SNPs) across the chromosomes. Table S2. The passport information of 109 watermelon accessions in the germplasm collection.

## References

[1] Food and Agriculture Organization FAOSTAT. http://www.fao.org/faostat/en/#data.

[2] Kousik C.S., Ikerd J.L., Mandal M. (2019). Relative Susceptibility of Commercial Watermelon Varieties to Powdery Mildew. Crop. Prot..

[3] Salcedo A., Parada-Rojas C.H., Guerrero R., Stahr M., D’Arcangelo K.N., McGregor C., Kousik C., Wehner T., Lina M., Dutta S.K., Nimmakayala P., Reddy U.K. (2023). The NLR Family of Disease Resistance Genes in Cultivated Watermelon and Other Cucurbits: Opportunities and Challenges. The Watermelon Genome.

[4] Tetteh A.Y., Wehner T.C., Davis A.R. (2013). Inheritance of Resistance to Powdery Mildew Race 2 in *Citrullus lanatus* var. lanatus. HortScience.

[5] Davis A.R., Levi A., Tetteh A., Wehner T., Russo V., Pitrat M. (2007). Evaluation of Watermelon and Related Species for Resistance to Race 1W Powdery Mildew. J. Am. Soc. Hort. Sci..

[6] Kim K.H., Hwang J.H., Han D.Y., Park M., Kim S., Choi D., Kim Y., Lee G.P., Kim S.T., Park Y.H. (2015). Major Quantitative Trait Loci and Putative Candidate Genes for Powdery Mildew Resistance and Fruit-Related Traits Revealed by an Intraspecific Genetic Map for Watermelon (*Citrullus lanatus* var. *lanatus*). PLoS ONE.

[7] Mandal M.K., Suren H., Kousik C. (2020). Elucidation of Resistance Signaling and Identification of Powdery Mildew Resistant Mapping Loci (*ClaPMR2*) during Watermelon-*Podosphaera Xanthii* Interaction Using RNA-Seq and Whole-Genome Resequencing Approach. Sci. Rep..

[8] Deng Y., Liu X., Liu S., Li X., Xue L., Bai T., Xu B., Li G., Sun Y., Zhang X. (2024). Fine Mapping of *ClLOX*, a QTL for Powdery Mildew Resistance in Watermelon (*Citrullus lanatus* L.). Theor. Appl. Genet..

[9] Wu S., Wang X., Reddy U., Sun H., Bao K., Gao L., Mao L., Patel T., Ortiz C., Abburi V.L. (2019). Genome of ‘Charleston Gray’, the Principal American Watermelon Cultivar, and Genetic Characterization of 1,365 Accessions in the U.S. National Plant Germplasm System Watermelon Collection. Plant Biotechnol. J..

[10] Korte A., Farlow A. (2013). The Advantages and Limitations of Trait Analysis with GWAS: A Review. Plant Methods.

[11] Sonah H., O’Donoughue L., Cober E., Rajcan I., Belzile F. (2015). Identification of Loci Governing Eight Agronomic Traits Using a GBS-GWAS Approach and Validation by QTL Mapping in Soya Bean. Plant Biotechnol. J..

[12] Ben-Naim Y., Cohen Y. (2015). Inheritance of Resistance to Powdery Mildew Race 1W in Watermelon. Phytopathology.

[13] Porterfield R., Meru G. (2017). Candidate Susceptibility Genes for Powdery and Downy Mildew in Watermelon and Squash. J. Phylogenetics Evol. Biol..

[14] Zhang Z., Ersoz E., Lai C.Q., Todhunter R.J., Tiwari H.K., Gore M.A., Bradbury P.J., Yu J., Arnett D.K., Ordovas J.M. (2010). Mixed Linear Model Approach Adapted for Genome-wide Association Studies. Nat. Genet..

[15] Huang M., Liu X., Zhou Y., Summers R.M., Zhang Z. (2018). BLINK: A Package for the next Level of Genome-wide Association Studies with Both Individuals and Markers in the Millions. Gigascience.

[16] Yu J., Buckler E.S. (2006). Genetic Association Mapping and Genome Organization of Maize. Curr. Opin. Biotechnol..

[17] Nimmakayala P., Levi A., Abburi L., Lakshmi Abburi V., Tomason Y.R., Saminathan T., Gopinath Vajja V., Malkaram S., Reddy R., Wehner T.C. (2014). Single Nucleotide Polymorphisms Generated by Genotyping by Sequencing to Characterize Genome-wide Diversity, Linkage Disequilibrium, and Selective Sweeps in Cultivated Watermelon. BMC Genom..

[18] The UniProt Consortium (2023). UniProt: The Universal Protein Knowledgebase in 2023. Nucleic Acids Res..

[19] García J.C., Guadagno A., Paytuvi-Gallart A., Saera-Vila A., Amoroso C.G., D’esposito D., Andolfo G., Aiese Cigliano R., Sanseverino W., Ercolano M.R. (2022). PRGdb 4.0: An Updated Database Dedicated to Genes Involved in Plant Disease Resistance Process. Nucleic Acids Res..

[20] Iovieno P., Andolfo G., Schiavulli A., Catalano D., Ricciardi L., Frusciante L., Ercolano M.R., Pavan S. (2015). Structure, Evolution and Functional Inference on the Mildew Locus O (MLO) Gene Family in Three Cultivated *Cucurbitaceae* spp.. BMC Genom..

[21] Yadav V., Wang Z., Guo Y., Zhang X. (2022). Comparative Transcriptome Profiling Reveals the Role of Phytohormones and Phenylpropanoid Pathway in Early-Stage Resistance against Powdery Mildew in Watermelon (*Citrullus lanatus* L.). Front. Plant Sci..

[22] Piepho H.P., Möhring J., Melchinger A.E., Büchse A. (2008). BLUP for Phenotypic Selection in Plant Breeding and Variety Testing. Euphytica.

[23] Choudhary H., Padmanabha K., Jat G.S., Behera T.K., Dutta S.K., Nimmakayala P., Reddy U.K. (2023). Challenges of Traditional Breeding in Watermelon. The Watermelon Genome.

[24] Guo T., Yang J., Li D., Sun K., Luo L., Xiao W., Wang J., Liu Y., Wang S., Wang H. (2019). Integrating GWAS, QTL, Mapping and RNA-Seq to Identify Candidate Genes for Seed Vigor in Rice (*Oryza sativa* L.). Mol. Breed..

[25] Elshire R.J., Glaubitz J.C., Sun Q., Poland J.A., Kawamoto K., Buckler E.S., Mitchell S.E. (2011). A Robust, Simple Genotyping-by-Sequencing (GBS) Approach for High Diversity Species. PLoS ONE.

[26] Hammond-Kosack K.E., Kanyuka K. (2007). Resistance Genes (*R* Genes) in Plants. Encyclopedia of Life Sciences.

[27] Freymark G., Diehl T., Miklis M., Romeis T., Panstruga R. (2007). Antagonistic Control of Powdery Mildew Host Cell Entry by Barley Calcium-Dependent Protein Kinases (CDPKs). Mol. Plant-Microbe Interact..

[28] Geng S., Li A., Tang L., Yin L., Wu L., Lei C., Guo X., Zhang X., Jiang G., Zhai W. (2013). *TaCPK2-A*, a Calcium-Dependent Protein Kinase Gene That Is Required for Wheat Powdery Mildew. J. Exp. Bot..

[29] Hu Y., Cheng Y., Yu X., Liu J., Yang L., Gao Y., Ke G., Zhou M., Mu B., Xiao S. (2021). Overexpression of Two *CDPK*s from Wild Chinese Grapevine Enhances Powdery Mildew Resistance in *Vitis Vinifera* and Arabidopsis. New Phytol..

[30] Qiu J.L., Zhou L., Yun B.W., Nielsen H.B., Fiil B.K., Petersen K., MacKinlay J., Loake G.J., Mundy J., Morris P.C. (2008). Arabidopsis Mitogen-Activated Protein Kinase Kinases MKK1 and MKK2 Have Overlapping Functions in Defense Signaling Mediated by MEKK1, MPK4, and MKS1^1[W]^. Plant Physiol..

[31] Rodriguez M.C.S., Petersen M., Mundy J. (2010). Mitogen-Activated Protein Kinase Signaling in Plants. Annu. Rev. Plant Biol..

[32] Zhang C., Anarjan M.B., Win K.T., Begum S., Lee S. (2021). QTL-Seq Analysis of Powdery Mildew Resistance in a Korean Cucumber Inbred Line. Theor. Appl. Genet..

[33] Tek M.I., Calis O. (2022). Mechanisms of Resistance to Powdery Mildew in Cucumber. Phytopathol. Mediterr..

[34] Kawasaki T., Koita H., Nakatsubo T., Hasegawa K., Wakabayashi K., Takahashi H., Umemura K., Umezawa T., Shimamoto K. (2006). Cinnamoyl-CoA Reductase, a Key Enzyme in Lignin Biosynthesis, Is an Effector of Small GTPase Rac in Defense Signaling in Rice. Proc. Natl. Acad. Sci. USA.

[35] Wang P., Guo L., Morgan J., Dudareva N., Chapple C. (2022). Transcript and Metabolite Network Perturbations in Lignin Biosynthetic Mutants of Arabidopsis. Plant Physiol..

[36] Derikvand M.M., Sierra J.B., Ruel K., Pollet B., Do C.T., Thévenin J., Buffard D., Jouanin L., Lapierre C. (2008). Redirection of the Phenylpropanoid Pathway to Feruloyl Malate in *Arabidopsis* Mutants Deficient for Cinnamoyl-CoA Reductase 1. Planta.

[37] Ramonell K., Berrocal-Lobo M., Koh S., Wan J., Edwards H., Stacey G., Somerville S. (2005). Loss-of-Function Mutations in Chitin Responsive Genes Show Increased Susceptibility to the Powdery Mildew Pathogen *Erysiphe cichoracearum*. Plant Physiol..

[38] Lin S.S., Martin R., Mongrand S., Vandenabeele S., Chen K.C., Jang I.C., Chua N.H. (2008). RING1 E3 Ligase Localizes to Plasma Membrane Lipid Rafts to Trigger FB1-Induced Programmed Cell Death in Arabidopsis. Plant J..

[39] Kawasaki T., Nam J., Boyes D.C., Holt B.F., Hubert D.A., Wiig A., Dangl J.L. (2005). A Duplicated Pair of Arabidopsis RING-Finger E3 Ligases Contribute to the RPM1- and RPS2-Mediated Hypersensitive Response. Plant J..

[40] Trujillo M., Ichimura K., Casais C., Shirasu K. (2008). Negative Regulation of PAMP-Triggered Immunity by an E3 Ubiquitin Ligase Triplet in *Arabidopsis*. Curr. Biol..

[41] Induri B., Nimmakayala P., Reddy U.K., Dutta S.K., Nimmakayala P., Reddy U.K. (2023). Genomic Resources for Disease Resistance in Watermelon. The Watermelon Genome.

[42] Doyle J.J., Hewitt G.M., Johnston A.W.B., Young J.P.W. (1990). DNA Protocols for Plants. Molecular Techniques in Taxonomy.

[43] Zheng Y., Wu S., Bai Y., Sun H., Jiao C., Guo S., Zhao K., Blanca J., Zhang Z., Huang S. (2019). Cucurbit Genomics Database (CuGenDB): A Central Portal for Comparative and Functional Genomics of Cucurbit Crops. Nucleic Acids Res..

[44] Perrier X., Jacquemoud-Collet J.P. DARwin Software. http://darwin.cirad.fr/darwin.

[45] Alexander D.H., Novembre J., Lange K. (2009). Fast Model-Based Estimation of Ancestry in Unrelated Individuals. Genome. Res..

[46] Paul G., Steven J., Darice G., Janine F., Nicholas D., Nathan B., Matthew B., Ty N., Michael W. PySAM (Python Wrapper for System Advisor Model “SAM”). http://github.com/NREL/pysam.

[47] Lipka A.E., Tian F., Wang Q., Peiffer J., Li M., Bradbury P.J., Gore M.A., Buckler E.S., Zhang Z. (2012). GAPIT: Genome Association and Prediction Integrated Tool. Bioinformatics.

[48] Cingolani P., Platts A., Wang L.L., Coon M., Nguyen T., Wang L., Land S.J., Ruden D.M., Lu X. (2012). A Program for Annotating and Predicting the Effects of Single Nucleotide Polymorphisms, SnpEff: SNPs in the Genome of *Drosophila Melanogaster* Strain *w*^1118^; Iso-2; Iso-3. Fly.

