# Supplementary material for: Identification of Genomic Regions Associated with Powdery Mildew Resistance in Watermelon through Genome-Wide Association Study
Source: Plants (Basel). 2024 Sep 27;13(19):2708. doi: 10.3390/plants13192708 (PMC11479075; doi:10.3390/plants13192708)
Supplement: Supplementary file 1 [file plants-13-02708-s001.zip › plants-3224678-supplementary.pdf]

plants

MDPI

Supplementary materials

Identification of Powdery Mildew Resistance-associated Genomic Regions in Watermelon through Genome-wide Association Study

Oak-Jin Lee [1,3,*], Koeun Han [1], Hye-Eun Lee [1], Hyo-Bong Jeong [2], Nari yu [1] and Won Byoung Chae [3]

[1] Vegetable Research Division, National Institute of Horticultural and Herbal Science, Rural Development Administration, Wanju 55365, Republic of Korea; ojlee6524@korea.kr(O.-J.L); hke1221@korea.kr(K.H); helee72@korea.kr(H.-E.L); ynr7328@korea.kr(N.Y)
[2] Research Management Division, Rural Development Administration, Jeonju 54875, Republic of Korea; bong9846@korea.kr
[3] Department of Environmental Horticulture, College of Bioresource Science, Dankook University, Cheonan 31116, Republic of Korea; wbchae75@dankook.ac.kr
* Correspondence: ojlee6524@korea.kr; Tel.: +82-63-238-6615

Supplementary table

Table S1. Distribution of single nucleotide polymorphisms (SNPs) across the chromosomes.

Chromosome	Number of SNP	Interval (Mb)	
		Average	Maximum
1	3,304	0.011	1.00
2	2,879	0.013	1.14
3	2,436	0.013	1.26
4	1,787	0.015	0.76
5	3,685	0.010	0.48
6	2,556	0.012	1.32
7	2,302	0.014	1.45
8	2,198	0.013	0.72
9	3,110	0.012	0.38
10	2,904	0.012	0.63
11	2,604	0.012	0.58
Total	29,765	-	-

Table S2. The passport information of 109 watermelon accessions in the germplasm collection.

Accession	Species	Origin country/region	Homozygosity (%)
PG001	*Citrullus lanatus*	Korea	99.56
PG002	*Citrullus lanatus*	Korea	99.71
PG003	*Citrullus lanatus*	Korea	98.91
PG004	*Citrullus lanatus*	Korea	99.43
PG005	*Citrullus lanatus*	Korea	99.18
PG006	*Citrullus lanatus*	Korea	99.77
PG007	*Citrullus lanatus*	Korea	99.42
PG008	*Citrullus lanatus*	Korea	94.07
PG009	*Citrullus lanatus*	Korea	99.13
PG011	*Citrullus lanatus*	Korea	99.72
PG012	*Citrullus lanatus*	Korea	99.55
PG013	*Citrullus lanatus*	Korea	99.37
PG014	*Citrullus lanatus*	Korea	99.49
PG015	*Citrullus lanatus*	Korea	98.81
PG016	*Citrullus lanatus*	Korea	99.37
PG017	*Citrullus lanatus*	Korea	99.47
PG018	*Citrullus lanatus*	Korea	99.55
PG019	*Citrullus lanatus*	Korea	99.32
PG020	*Citrullus lanatus*	United States	99.35
PG021	*Citrullus mucosospermus*	Nigeria	91.94
PG022	*Citrullus amarus*	Democratic Republic of the Congo	97.51
PG023	*Citrullus amarus*	Zimbabwe	95.84
PG024	*Citrullus lanatus*	Unknown	96.79
PG026	*Citrullus lanatus*	Vietnam	99.54
PG027	*Citrullus lanatus*	Korea	99.53
PG028	*Citrullus amarus*	South Africa	96.75
PG029	*Citrullus lanatus*	United States	99.67
PG030	*Citrullus amarus*	Korea	99.32
PG031	*Citrullus lanatus*	South Africa	97.38
PG032	*Citrullus lanatus*	Zambia	99.39
PG033	*Citrullus amarus*	Botswana	96.78
PG034	*Citrullus amarus*	Zimbabwe	97.25
PG035	*Citrullus amarus*	South Africa	97.59
PG036	*Citrullus lanatus*	Zambia	97.51
PG037	*Citrullus amarus*	Namibia	96.69
PG038	*Citrullus amarus*	South Africa	96.20
PG040	*Citrullus lanatus*	United States	99.62
PG041	*Citrullus amarus*	South Africa	97.41
PG042	*Citrullus amarus*	Zimbabwe	97.44
PG043	*Citrullus lanatus*	Mexico	99.32
PG044	*Citrullus amarus*	Zimbabwe	97.58
PG045	*Citrullus amarus*	Zimbabwe	97.81
PG046	*Citrullus lanatus*	South Africa	97.45
PG047	*Citrullus lanatus*	Korea	99.29
PG048	*Citrullus lanatus*	Korea	99.53
PG049	*Citrullus lanatus*	Korea	99.36
PG050	*Citrullus lanatus*	Korea	99.41
PG051	*Citrullus lanatus*	Korea	99.61

PG052	*Citrullus lanatus*	Korea	99.29
PG053	*Citrullus lanatus*	Korea	99.65
PG054	*Citrullus lanatus*	Korea	99.55
PG055	*Citrullus lanatus*	Korea	99.52
PG056	*Citrullus lanatus*	Korea	99.57
PG057	*Citrullus lanatus*	Korea	98.69
PG059	*Citrullus lanatus*	Korea	99.28
PG060	*Citrullus lanatus*	Korea	99.54
PG061	*Citrullus lanatus*	Korea	99.59
PG062	*Citrullus lanatus*	Korea	99.03
PG063	*Citrullus amarus*	Zimbabwe	96.50
PG064	*Citrullus amarus*	Botswana	97.80
PG065	*Citrullus lanatus*	Korea	99.48
PG066	*Citrullus lanatus*	United States	99.70
PG067	*Citrullus amarus*	South Africa	93.58
PG068	*Citrullus lanatus*	Korea	99.43
PG069	*Citrullus lanatus*	United States	99.22
PG070	*Citrullus amarus*	Zimbabwe	94.47
PG071	*Citrullus lanatus*	Syria	99.63
PG072	*Citrullus amarus*	South Africa	97.11
PG073	*Citrullus amarus*	Zimbabwe	92.52
PG074	*Citrullus lanatus*	Botswana	97.13
PG075	*Citrullus lanatus*	Netherlands	99.67
PG076	*Citrullus lanatus*	United States	99.58
PG077	*Citrullus amarus*	Zimbabwe	96.40
PG078	*Citrullus lanatus*	China	99.16
PG079	*Citrullus lanatus*	Turkey	99.63
PG080	*Citrullus lanatus*	China	99.49
PG081	*Citrullus lanatus*	Vietnam	99.63
PG082	*Citrullus lanatus*	Indonesia	98.89
PG083	*Citrullus lanatus*	China	99.41
PG084	*Citrullus lanatus*	Korea	99.20
PG085	*Citrullus lanatus*	China	99.65
PG086	*Citrullus lanatus*	Korea	99.16
PG087	*Citrullus lanatus*	China	99.22
PG089	*Citrullus lanatus*	Zambia	99.60
PG090	*Citrullus lanatus*	Zambia	99.44
PG091	*Citrullus amarus*	Zambia	93.73
PG092	*Citrullus amarus*	Zimbabwe	97.24
PG093	*Citrullus amarus*	Zimbabwe	97.04
PG094	*Citrullus amarus*	Zimbabwe	97.78
PG095	*Citrullus amarus*	Zambia	95.42
PG096	*Citrullus lanatus*	Zambia	99.51
PG097	*Citrullus lanatus*	Turkey	99.42
PG098	*Citrullus lanatus*	Zimbabwe	99.27
PG099	*Citrullus lanatus*	Zimbabwe	98.08
PG100	*Citrullus lanatus*	Turkey	98.84
PG101	*Citrullus lanatus*	Angola	99.72
PG102	*Citrullus lanatus*	Angola	99.61
PG103	*Citrullus lanatus*	Nigeria	99.52
PG104	*Citrullus lanatus*	Zimbabwe	99.60

PG105	*Citrullus lanatus*	Zimbabwe	99.61
PG106	*Citrullus lanatus*	India	99.76
PG108	*Citrullus amarus*	Zimbabwe	96.88
PG118	*Citrullus lanatus*	Zimbabwe	99.65
PG119	*Citrullus lanatus*	Zimbabwe	99.04
PG121	*Citrullus lanatus*	Zambia	99.72

Supplementary figure

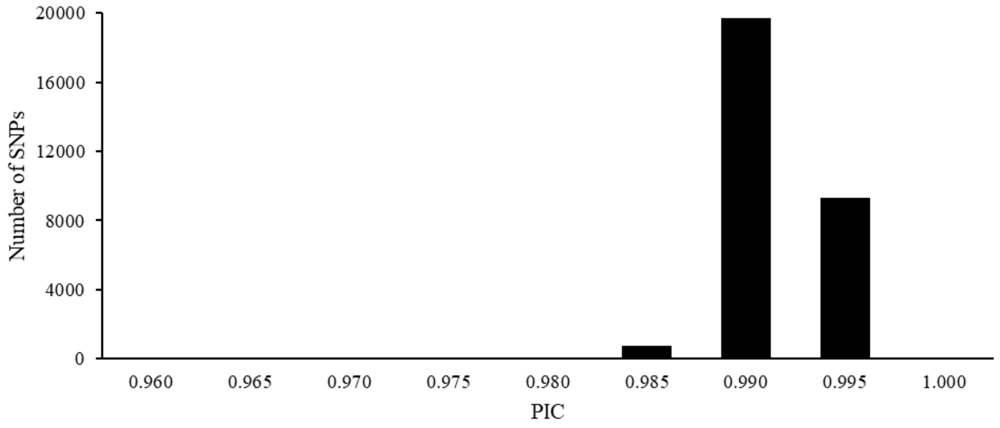

Figure S1. Distribution of polymorphic information content (PIC) across single nucleotide polymorphisms (SNPs).

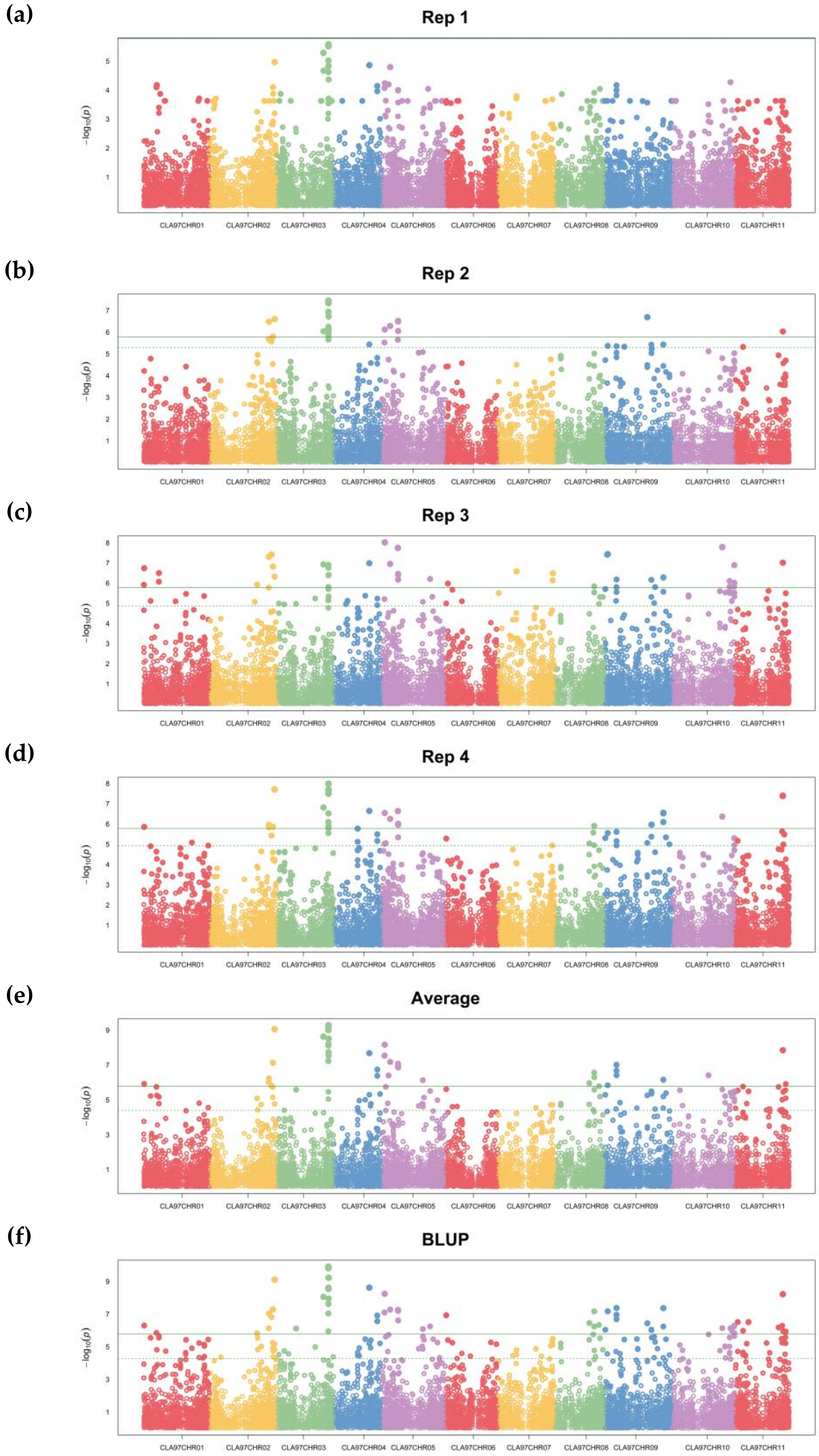

Figure S2. Manhattan plot from genome-wide association study (GWAS) using generalized linear model (GLM) for four replicates (a-d), averages of replicates (e) and best linear unbiased prediction (BLUP) values (f) of each accession in powdery mildew resistance. The horizontal solid and dashed lines indicate genome-wide and suggestive significance thresholds, respectively.
